# Association of Dermatoglyphic Peculiarities with Dental Caries in Preschool Children of Lucknow, India

**DOI:** 10.5005/jp-journals-10005-1331

**Published:** 2016-04-22

**Authors:** Ekta Singh, Sabyasachi Saha, GV Jagannath, Sanjay Singh, Sonali Saha, Nishita Garg

**Affiliations:** 1 Senior Lecturer, Department of Public Health Dentistry, Sardar Patel Post Graduate Institute of Dental and Medical Sciences, Lucknow Uttar Pradesh, India; 2 Professor and Head, Department of Public Health Dentistry, Sardar Patel Post Graduate Institute of Dental and Medical Sciences, Lucknow Uttar Pradesh, India; 3 Reader, Department of Public Health Dentistry, Sardar Patel Post Graduate Institute of Dental and Medical Sciences, Lucknow Uttar Pradesh, India; 4 Lecturer, Department of Public Health Dentistry, Sardar Patel Post Graduate Institute of Dental and Medical Sciences, Lucknow Uttar Pradesh, India; 5 Reader, Department of Pedodontics and Preventive Dentistry, Sardar Patel Post Graduate Institute of Dental and Medical Sciences Lucknow, Uttar Pradesh, India; 6 Lecturer, Department of Pedodontics and Preventive Dentistry, Sardar Patel Post Graduate Institute of Dental and Medical Sciences Lucknow, Uttar Pradesh, India

**Keywords:** Dental caries, Dermatoglyphics, Fingerprint pattern, Preschool children.

## Abstract

**Background:** Dermatoglyphics refers to study of the intricate dermal ridge configurations on the skin covering the palmar and plantar surfaces of hand and feet. The basis of considering dermatoglyphic patterns as genetic marker for dental caries is that the epithelium of finger buds as well as enamel has ectodermal origin, and both develop at the same time of intrauterine life.

**Aim:** To assess the relationship between fingerprint patterns and dental caries among preschool children of Lucknow city.

**Study design:** This study was of cross-sectional design.

**Materials and methods:** The study group comprised 512 preschool children 2-6 years of age. The prevalence of caries was recorded using "Dentition status and treatment needs" (WHO basic oral health assessment form, 1997). They were divided into three groups as follows: Group I (dmft score = 0-2), group II (dmft score = 3-4) and group III (dmft score ≥5). The handprints of each child were taken using a stamp pad. The fingertip patterns were analyzed according to the classical method and were classified according to the topological method. The frequency of occurrence of type of dermatoglyphic pattern on fingertip of each digit was noted.

**Statistics:** Chi-square test was used to test the significant difference in proportions. Means were compared using Student’s t-test and analysis of variance (ANOVA) or F-test.

**Results:** Subjects belonging to groups II and III showed maximum occurrence of whorl pattern on all digits. Group I subjects had maximum occurrence of arch pattern. All the variables had statistically significant value, with a degree of divergence of specific dermatoglyphic patterns among all three groups.

**Conclusion:** The dental caries susceptibility of an individual increased with incidence of whorl pattern and it decreased with incidence of arch pattern.

**How to cite this article:** Singh E, Saha S, Jagannath GV, Singh S, Saha S, Garg N. Association of Dermatoglyphic Peculiarities with Dental Caries in Preschool Children of Lucknow, India. Int J Clin Pediatr Dent 2016;9(1):39-44.

## INTRODUCTION

Dermatoglyphics as coined by Cummins et al^1^ refers to study of the intricate dermal ridge configurations on the skin covering the palmar and plantar surfaces of hand and feet. Dermatoglyphics is the art and science of studying the patterns of fingerprints.^2^ Fingerprints are found in humans and some animals. They are unique to all individuals and remain unchanged over the lifetime. For centuries, the features of the hands have fascinated scholars, sages, theologians, doctors and layman alike. Rather through decades of scientific research, the hands have come to be recognized as a powerful tool in the diagnosis of psychosocial, medical and genetic conditions.^3^

The dermal ridges take their origin from fetal volar pads that appear in the 6th-7th week of embryonic life, i.e., at the same time as that of tooth formation in intraembryonic life. This means that the genetic message contained in the genome (normal or abnormal) is deciphered during this period and is also reflected by dermatoglyphics.^4^ These volar pads occur as mound-shaped elevations of the mesenchymal tissue situated above the proximal end of most distal metacarpal bone on each finger, in each interdigital area. The size and position of these volar pads, to a large extent, are responsible for the type of configuration of ridge patterns.^5^ The ridge patterns are completed by the 12th-14th week of gestation, i.e., at the same time as that of tooth formation completion in intraembryonic life. Both primary genetic determination and development secondary to flexion function have been suggested as the mechanisms of underlying the crease development.

The basis of considering dermatoglyphic patterns as genetic marker for dental caries is that the epithelium of finger buds and enamel have ectodermal origin, and both develop at the same time of intrauterine life.^6^

Sir Francis Galton,^5^ in 1892, gave the basic nomenclature of the types of fingerprint patterns. They are grouped as loops, whorls and arches. The loops can be further subdivided into ulnar loops and radial loops.

Studies have shown that dental caries susceptibility of an individual increased with incidence of whorl pattern and decreased with incidence of loop pattern.^3,4^

The dermatoglyphic patterns may be utilized effectively to study the genetic basis of dental caries. In a developing country like India, it might prove to be a noninvasive, inexpensive and effective tool for screening.^3^

It is also convenient, cost-effective and requires no hospitalization. It can help in predicting the phenotype of a possible future health condition. There are sparse studies of dermatoglyphic findings in children of Indian population with dental caries. Hence, the aim of the study was to assess the relationship between fingerprint patterns and dental caries among preschool children of Lucknow city.

## MATERIALS AND METHODS

### Study Design

A cross-sectional study was undertaken comprising of 512 preschool children 2-6 years of age.

### Groups

**Table:** 

*Sl. no.*		*Groups*		*dmft score*	
1		I		0-2	
2		II		3-4	
3		III		5 or more	

### Source of Data and Study Population

The subjects for this study were chosen from different private/government playgroup, nursery and kindergarten schools of Lucknow city. The sample was selected by multistage cluster random sampling technique.

In the first stage, Lucknow city was divided geographically into five areas, i.e., East, West, North, South and Central. Approximately 22 wards came under each of these geographic areas. In the second stage, one ward was randomly selected from each geographic area. The list of all the wards from the five geographic areas was obtained from Census Enumeration Areas Data. In the third stage, survey was conducted in five preschools and primary schools and 102, 104, 101, 103, 102 children from 1st, 2nd, 3rd, 4th and 5th wards were selected, respectively, to attain a total sample of 512.

### Inclusion Criteria

- All the children 2-6 years of age who were present in class along with their mothers on the day of survey.

### Exclusion Criteria

- Subjects with special health care needs (e.g., cleft lip and palate syndromes, medically and physically challenged).
- Children who are undergoing or who had undergone orthodontic treatment.
- Those children who did not cooperate during the examination procedure.

### Informed Consent and Ethical Clearance

A written informed consent was obtained from the parents of subjects. This study was reviewed by the Institutional Ethical Committee of Sardar Patel Post Graduate Institute of Dental & Medical Sciences, Lucknow and necessary approval was obtained.

### Calibration

Before the start of the study, the investigator was calibrated, 50 preschool children who possessed dental caries were selected and examined. The recorder in the study was also previously trained in the department regarding the proper method of fingerprint recording. By comparing the results of the two examinations, the examiner was able to obtain an estimate of the extent and nature of the diagnostic variability and the Kappa (κ) coefficient was estimated to be >0.8.

### Survey Procedure

Single examiner interviewed and examined the subjects. In each school, the available subjects belonging to specific age group were examined by the examiner by making each subject sit on a small chair with a backrest. The recorder was seated in front of the subject close to the examiner. The position enables the examiner to confirm that the finding was accurately recorded. The examiner counterchecked the entries made by the recorder at the end of each examination.

Clinical assessment of dental caries was done by using dentition status and treatment need (WHO basic oral health proforma, 1997) for deciduous teeth.

### Recording of Handprints

The methodology of taking handprints was explained to the children and they were cautioned not to smear the dye on their body or clothing.

- The hands of the patients were scrubbed thoroughly and blot dried.
- The duplicating ink was dispensed in a pea-sized amount for each hand and spread to the entire area of palm and fingers with the help of a gauze pack. It was important that a very minimal amount of dye was taken as this helped in getting clear handprints. The more the amount of dye, darker were the prints, and thus unreadable. Various dyes were tried before settling for blue duplicating ink.
- Once even spread of the dye was ensured, the patient was asked to place his/her hand with all fingers apart on a sheet of paper. If the patient shook his/her hand during the procedure, the recording got smudged. Light pressure was applied over all the fingers to ensure proper recording of prints.
- The handprints obtained were checked for their clarity through a magnifying glass (2x) and a number was given to it. The presence of core and the triradii of the pattern were noted to include the handprint in the study. If these landmarks cannot be demarcated clearly, then taking another handprint was recommended. The handprints taken were preserved with caution. A single-blinded examiner observed all the handprints.
- Often, it was noted that the thumb did not provide proper prints, which could be due to its spatial orientation as compared to the rest of the fingers, so a separate impression of the thumb was taken.

### Infection Control

Disposable mouth masks and gloves were used by the examiner during examination. Autoclaved clinical examination instruments of 40 sets were carried for clinical examination.

### Statistical Analysis

The data were entered into Microsoft Excel XP software program. Statistical analysis was done by Statistical Package for the Social Sciences (SPSS) software package (SPSS 16 Inc, Chicago IL, USA). The values obtained were statistically analyzed with the Chi-square test, Fisher’s exact test, Student’s t-test and analysis of variance (ANOVA) and any other suitable methods for correlating the fingerprint pattern with dental caries among the study population.

## RESULTS

**Graph 1:**
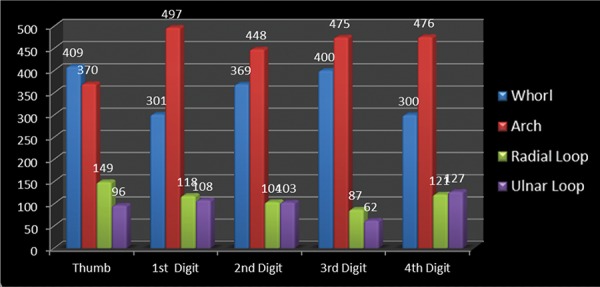
Distribution of dermatoglyphic patterns in all digits in total of left and right sides

**Graph 2:**
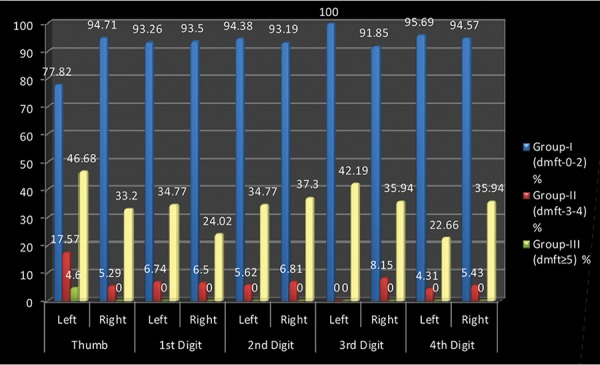
Distribution of arch pattern in relation to caries experience

**Graph 3:**
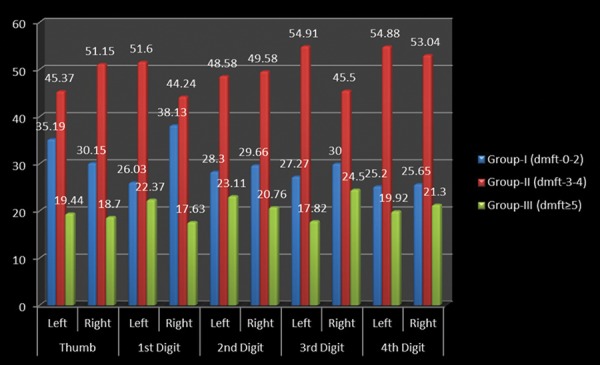
Distribution of whorl pattern in relation to caries experience

**Graph 4:**
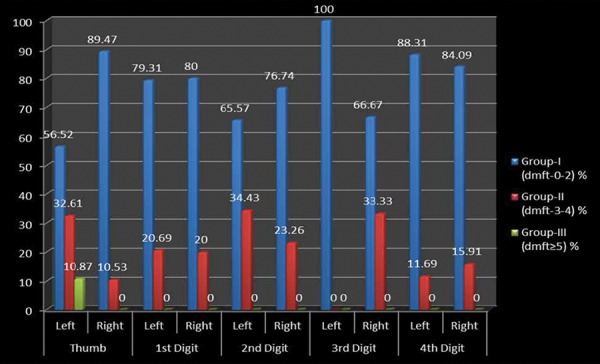
Distribution of ulnar loop pattern ir relation to caries experience

**Graph 5:**
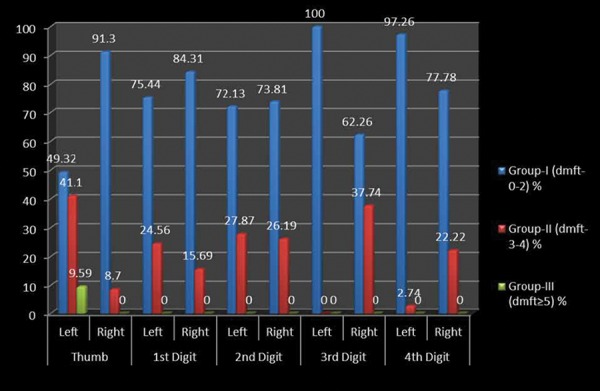
Distribution of radial loop pattern in relation to caries experience

A cross-sectional study was undertaken comprising of 512 preschool children 2-6 years of age to assess the relationship between fingerprint patterns and dental caries among preschool children of Lucknow city. Table 1 shows the distribution of the study subjects according to age and gender. A total of 512 children comprising of 243 (47.46%) males and 269 (52.54%) females were examined. Majority of males (106; 20.7%) and females (110; 21.48%) were 3 years of age. Table 2 shows the genderwise distribution of the children according to early childhood caries experience. Statistically significant difference in prevalence of early childhood caries was found between males and females (p = 0.04826). Though males had a higher mean dmft score (1.97 ± 2.01) as compared with females (1.75 ± 2.20), the difference was not statistically significant (p = 0.2480). On evaluating the distribution of the study subjects according to dermatoglyphic patterns on the left and right sides, it was revealed that a total of 409, 370, 149 and 96 children had whorl, arch, radial loop and ulnar loop on thumb respectively. Overall arch pattern was most prominent followed by whorl, radial loop and ulnar loop (Graph 1). Table 3 shows the age and genderwise distribution of the study subjects according to different caries experience. Across all age groups, majority of children belonged to group I (dmft 0-2) followed by group II (dmft 3-4) and group III (dmft ≥ 5). Graph 2 shows the distribution of the study subjects according to arch pattern. Majority of the children (156, 77.82%; 161, 94.71%) with arch pattern on left and right thumb belonged to group I (dmft 0-2). On the left and right 1st digit, maximum children (166, 93.26%; 115, 93.5%) with arch pattern belonged to group I (dmft 0-2). Similarly, the arch pattern on left and right 2nd, 3rd and 4th digit (168, 94.38%; 178, 93.19%; 216, 100%; 169, 91.85%; 111, 95.69%; 174, 94.57%) children, respectively, belonged to group I (dmft 0-2). Graph 3 shows the distribution of the study subjects according to whorl pattern. Majority of the children (49, 45.37%; 134, 51.15%) with whorl pattern on left and right thumb belonged to group II (dmft 3-4). In the left and right 1st digit, maximum children (113, 51.15%; 123, 44.24%) with whorl pattern belonged to group II (dmft 3-4), and similarly, the whorl pattern on left and right 2nd, 3rd and 4th digit (103, 48.58%; 117, 49.58%; 151, 54.91%; 91, 45.5%; 135, 54.88%; 122, 53.04%) children, respectively, belonged to group II (dmft 3-4). Graph 4 shows the distribution of the study subjects according to ulnar loop pattern. Majority of the children (52, 56.52%; 51, 89.47%) with ulnar loop on left and right thumb belonged to group I (dmft 0-2). In the left and right 1st digit, maximum children (46, 79.31%; 48, 80%) with ulnar loop pattern belonged to group I (dmft 0-2) and similarly the ulnar loop pattern on left and right 2nd, 3rd and 4th digit (40, 65.57%; 33, 76.74%; 12, 100%; 50, 66.67%; 68, 88.31%; 37, 84.09%) children, respectively, belonged to group I (dmft 0-2). Graph 5 shows the distribution of the study subjects according to radial loop pattern. Majority of the children (36, 49.32%; 21, 91.3%) with radial loop on left and right thumb belonged to group I (dmft 0-2). In the left and right 1st digit, maximum children (43, 75.44%; 43, 84.31%) with radial loop pattern belonged to group I (dmft 0-2) and similarly the radial loop pattern on left and right 2nd, 3rd and 4th digit (44, 72.13%; 31, 73.81%; 9, 100%; 33, 62.26%; 71, 97.26%; 42, 77.78%) children, respectively, belonged to group I (dmft 0-2).

**Table 1:** **Table 1:** Age and genderwise distribution of the children

*Age (in years)*		*Gender*		*N*		*Percentage*	
2		M		10		1.95	
		F		22		4.30	
3		M		106		20.70	
		F		110		21.48	
4		M		43		8.40	
		F		56		10.94	
5		M		46		8.98	
		F		46		8.98	
6		M		38		7.42	
		F		35		6.84	
Total		M		243		47.46	
		F		269		52.54	

M: Male; F: Female

**Table 2:** **Table 2:** Genderwise distribution of the children according to early childhood caries experience

*Gender*		*Caries free* *N (%)*		*With dental* *caries N (%)*		Total		*dmft mean* *± SD*	
M		99 (40.74)		144 (59.26)		243		1.97 ± 2.01	
F		133 (49.44)		136 (50.56)		269		1.75 ± 2.20	
Total		232 (45.31)		280 (54.69)		512		1.85 ± 2.11	
						t-value = 1.1566			
						p = 0.2480			

Chi-square = 3.9010, df=1, p = 0.04826; SD: Standard deviation

**Table 3:** **Table 3:** Age and genderwise distribution of the children in relation to their caries experience

*Age* *(years)*		Gender		*Group I* *(dmft 0-2)*				*Group II* *(dmft 3-4)*				*Group III* *(dmft ≥5)*			
				*N*		%		*N*		*%*		*N*		*%*	
2		M		8		80.0		0		0.0		2		20.0	
		F		19		86.4		0		0.0		3		13.6	
Yates-corrected Chi-square = 0.0001; p = 1.0000															
3M		55		51.9		39		36.8		12		11.3	
		F		67		60.9		34		30.9		9		8.2	
Chi-square = 1.8748; p = 0.3914															
4M		24		55.8		10		23.3		9		20.9	
		F		36		64.3		6		10.7		14		25.0	
Chi-square = 2.8219; p = 0.2143															
5M		29		63.0		17		37.0		0		0.0	
		F		29		63.0		17		37.0		0		0.0	
Chi-square = 0.00001; p = 1.0000															
6		M		23		60.5		15		39.5		0		0.0	
		F		22		62.9		13		37.1		0		0.0	
Chi-square = 0.0442; p = 0.8348															
Total		M		139		57.2		81		33.3		23		9.5	
		F		173		64.3		70		26.0		26		9.7	

Chi-square = 3.3796; p = 0.1857; M: Male; F: Female

## DISCUSSION

For ages, the features of the hands have fascinated scholars, sages, theologians, doctors and laymen alike. The modern study of the hand is far removed from the popular image of the traditional palmist uttering mysterious incantations in an arcane language.^4^ Rather, through decades of scientific research, the hand has come to be recognized as a powerful tool in the diagnosis of psychological, medical and genetic conditions. Dermatoglyphics is considered as a window of congenital abnormalities and is a sensitive indicator of intrauterine anomalies.

Dental caries is also considered as a chronic complex, multifactorial disease for which a multitude of aetiologies like host and environmental factors have been proposed. The relative roles of heredity and environment (nature *vs* nurture) in the pathogenesis of dental caries have intrigued clinical and basic researchers for decades. There are numerous host resistance and risk factors for dental caries that are genetically determined. It is critical to realize that genes and environment do not act independently of each other. The type of fingerprint is unique and is based on the genetic characteristics of each individual. These dermal patterns once formed remain constant throughout life.

We infer the following from the findings of the study:

- Overall arch pattern was most prominent followed by whorls, radial loop and ulnar loop.
- Arches on all digits are considered to be less susceptible to dental caries.
- Increased occurrence of whorls was noted in the children showing comparatively higher df score.

In the present study, a sample of 512 preschool children was selected. Almost similar sample size of 550 children was examined in a study by Nidhi et al.^4^ A higher sample size of 1,250 children were examined in a study by Abhilash et al^3^ in which 47.46% were males and 52.54% were females. In a study conducted by Nidhi et al,^4^ the number of males and females was almost similar.

This study depicted that dental caries susceptibility of an individual increases with an increase in the incidence of whorl pattern. Similar findings were reported in studies conducted by. In the study by Abhilash et al, dental caries susceptibility of an individual increases with an increase in the incidence of whorl pattern (83%) and it was decreased with incidence of loop pattern and in the study by Nidhi et al^4^ the result showed that the caries group showed maximum occurrence of whorls, which were more prevalent in females on the left 3rd digit than in males where the whorls were found on the right hand 3rd digit and also low total ridge count, especially males.

The genetic susceptibility and added environmental factors the proneness for caries due to abnormality in the tooth structures like alteration in dental hard tissues like structure of dental enamel, tooth eruption and development may be reflected in the dermatoglyphics namely whorl and loop patterns.^7,8^

Hence, dermatoglyphics could indicate a genetic susceptibility to dental caries. In recent decades, a considerable improvement has been achieved in the concept of correlation between the types of pattern of lines on the fingers and some individual disorders.^9^ The pattern of lines on the fingers of the hand has been documented in medicine as a method of diagnosis.^10^

Numerous studies have described a potential genetic contribution to the risk for dental caries. There are numerous familial, pedigree and twin studies on dental caries. Studies on twins have provided strong evidence for the role of inheritance. So, the most convincing data on the role of genetics in the pathogenesis of dental caries have been developed by analyzing the caries incidence in monozygotic and dizygotic twins.^6^ It was also suggested by different studies that the children showed a remarkable similarity in dental caries to the susceptibility of the parents.^11,12^

Individuals with high resistance to dental caries had a specific immunoglobulin within saliva conveying immunity by lysing the cariogenic bacterial cells. It was suggested that this phenotype was inherited and transmitted as an autosomal dominant trait.^8^ Several reports and studies have also shown significant heritability for several microorganisms, including streptococci. Thus, genes and genetic abnormalities that lead to abnormal structural organization of teeth and its environment result in increased susceptibility to dental caries.^13^

Hence, we can also conclude susceptibility to dental caries has genetic control and this control could be multifactorial in nature.

## CONCLUSION

The dermatoglyphic patterns may be utilized effectively to study the genetic basis of dental caries. In a developing country like India, it might prove to be a noninvasive, inexpensive and effective tool for screening. These patterns may represent the genetic makeup of an individual and, therefore, his/her predisposition to certain diseases.

Given the expenses involved in conducting the analysis of the chromosomes themselves, dermatoglyphics can prove to be an extremely useful tool for preliminary investigations. The patterns seen in the form of ‘dermato-glyphics’ might play a significant role in the near future not only for the purpose of screening but also for studying the behavior of dental caries.

## References

[1] Cummins H, Keith HH, Midlo C, Montgomery RB, Wilder HH, Wilder IW (1929). Revised methods of interpretation and formulation of palmar dermatoglyphics. Am J Phys Anthropol.

[2] Bhargava SS, Sathawane RS (2012). Dermatoglyphics-exploring newer dimensions in diagnosis. Central India J Dent Sci.

[3] Abhilash PR, Divyashree R, Patil SG, Gupta M, Chandrasekar T, Karthikeyan R (2012). Dermatoglyphics in patients with dental caries: a study on 1250 individuals. J Contemp Dent Pract.

[4] Nidhi M, Arun R, Neeti B (2011). Palmistry: a tool for dental caries prediction. Indian J Dent Res.

[5] Galton F (1992). Fingerprints..

[6] Sharma A, Somani R (2009). Dermatoglyphic interpretation of dental caries and its correlation to salivary bacteria interactions: an *in vivo* Study. J Indian Soc Pedod Prev Dent.

[7] Hassel TM, Harris EL (1995). Genetic influences in caries and periodontal diseases. Crit Rev Oral Biol Med.

[8] Bretz WA, Schork NJ, Robinson MT, Coelho M, Costa S, Melo Filho MR, Weyant RJ, Hart TC (2005). Longitudinal analysis of heritability for dental caries traits. J Dent Res.

[9] Mulvihill JJ, Smith DW (1969). The genesis of dermatoglyphics. J Pediatr.

[10] Popich GA, Smith DW (1970). The genesis and significance of digital and palmar hand creases, preliminary report. J Pediatr.

[11] History of Dematoglyphics. Available at:. Fingerprints.net.com.

[12] Penrose LS (1965). Dermatoglyphic topology. Nature.

[13] Walkers NF (1957). The use of dermal configurations in the diagnosis of mongolism. J Pediatr.

